# Relationship Between Psychological Empowerment and Nurses’ Job Satisfaction: A Systematic Review and Correlational Meta-Analysis

**DOI:** 10.7759/cureus.58228

**Published:** 2024-04-14

**Authors:** Anees Hjazeen, Soha Kannan, Hindya O Al-Maqableh, Samar Maitah, Maha Abu-Radwan, Malak Sabbah

**Affiliations:** 1 Department of Biostatistics, Royal Medical Services, Amman, JOR; 2 Health Policy and Nursing, Directorate of Royal Medical Services Journal, Amman, JOR; 3 Health Services Administration, Faculty of Medicine, Yarmouk University, Irbid, JOR; 4 Queen Rania Al-Abdullah Hospital for Children, Royal Medical Services, Amman, JOR; 5 Princess Muna College of Nursing, Mutah University, Amman, JOR; 6 Department of Community Medicine, University of Jordan, Amman, JOR

**Keywords:** nurses, nursing, satisfaction, workplace, job, psychological empowerment

## Abstract

Psychological empowerment is a motivational concept that encompasses a person’s thoughts and perceptions that give a sense of behavior and commitment to the work. Psychological empowerment is widely acknowledged to be associated with nurses’ job satisfaction. However, this relationship has been found to be controversial. Therefore, this systematic review and meta-analysis aimed to investigate the relationship between psychological empowerment and nurses’ job satisfaction.

The electronic databases CINAHL, PubMed, Web of Science, and Google Scholar were utilized to search for relevant studies published from 2001 to 2024. The correlation coefficients were extracted for each eligible study and transformed into Fisher’s Z. Then, the pooled effect size (r coefficient) was computed using Fisher’s Z and the corresponding standard error. Moreover, I^2^ was used to assess the heterogeneity of studies. Begg’s rank and Egger’s test were employed to assess the publication bias. Sensitivity analysis was utilized to measure the robustness of study findings using the one-leave-out approach, and a critical appraisal tool for cross-sectional studies was adopted to assess the quality of included studies.

A total of 18 studies were selected for analysis with a total sample of 6,353 nurses from different countries. The included studies ranged from moderate to high quality based on the quality assessment checklist. The pooled effect size for the correlation between psychological empowerment and nurses’ job satisfaction was 0.512 (95% confidence interval = 0.406-0.604) with mild-to-moderate heterogeneity. Moreover, the majority of the studies confirmed a positive relationship between the two measured concepts.

This study presents evidence indicating that psychological empowerment has a sensible relationship with nurses’ job satisfaction. Therefore, nurse administrators should implement tailored strategies to trigger nurses’ psychological empowerment, aiming to boost job satisfaction and reduce turnover and burnout. However, additional studies are essential to establish a causal relationship.

## Introduction and background

Psychological empowerment is a cognitive and attitudinal mental state that assists nurses in feeling competent to perform their assigned tasks. It is the most effective strategy to improve productivity, use available resources within the organization, and release potential capabilities toward achieving the goals [1,2]. The nature of the nursing profession is emotionally exhausting due to workload, shortage of workforce, and unavailability of resources that can ultimately aggravate psychological and physical health problems and could influence patient care and job satisfaction [3]. However, psychological empowerment has been found to have an important role in employee retention, enhanced self-confidence, increased organizational effectiveness, work satisfaction, and reduced emotional burnout [4,5].

According to the literature, the relationship between psychological empowerment and nurses’ satisfaction is controversial [6,7]. This was confirmed by a meta-analysis conducted by Li et al. [8], who found a link between psychological empowerment and job satisfaction. Conversely, other studies reported that psychological empowerment had no relationship with nurses’ job satisfaction. Hence, conducting a meta-analysis, which involves aggregating the effect sizes from multiple studies, can systematically provide convincing and robust evidence regarding the nature of this relationship. Therefore, this systematic review and meta-analysis aimed to investigate the relationship between psychological empowerment and nurses’ job satisfaction.

## Review

Methodology

Search Strategy and Study Identification

This systematic review is reported according to the Preferred Reporting Items for Systematic Reviews and Meta-Analysis (PRISMA) protocol. An extensive literature search was performed from 2001 to 2024 across four databases (CINAHL, PubMed, Web of Science, and Google Scholar). The boolean operators were used to extract relevant articles with no time limitation for the search. The required articles were identified using numerous keywords, including “psychological empowerment, job, workplace satisfaction, nursing, and nurses.”

Inclusion Criteria

Studies were included if they met the following inclusion criteria: (I) correlational studies published in the English language irrespective of the geographical location; (II) studies conducted among nurses and not other healthcare providers; (III) studies with available full texts; (IV) primary studies providing statistics on the correlation coefficients between psychological empowerment and job satisfaction. The exclusion criteria involved studies presented as case reports, conference abstracts, educational presentations, or letters to editors. Two reviewers (SK, HM) selected the studies based on the aforementioned eligibility criteria, and any discrepancies were resolved by the principal author (AH).

Data Extraction

Three independent investigators performed data extraction (AH, SK, MA) and two investigators performed cross-validation (SM, MS). The last name of the first author, year of publication, place of study, sample size, study instrument with reliability measure, and correlation coefficients were extracted for each included study. Data extraction was executed using the Zotero citation manager.

Quality Assessment

Two investigators (MA, MS) independently assessed the methodological quality of the included correlational studies relying on the quality assessment tool for observational studies derived from Nguyen et al. [13], which includes five checklist items (the objective clearly described, the sampling method described, the period and location of the study clearly stated, proper examination method and procedure clearly pointed out, the samples clearly classified into different subgroups). A score of 2 is assigned for yes, 0 for no, and 1 for unsure. The scores range from 0 to 10 points, which are then multiplied by 100. A score of 0-40 indicates poor quality, 50-70 indicates medium quality, and 80-100 indicates high quality. Additionally, the quality assessments for the included studies were cross-checked by two investigators (SM, SK) (Table 1).

**Table 1:** The quality assessment of included studies.

Authors and publication year	Was the research objective clearly described and stated?	Was the sampling method described in detail?	Was the period and location of the study clearly stated?	Were the examination method and procedure clearly pointed out?	Were the samples clearly classified into different subgroups?	Total score (%)
Larrabee et al., 2003 [6]	2	2	0	2	2	80
Laschinger et al., 2004 [7]	2	2	2	2	0	80
Boamah et al., 2017 [14]	2	2	2	2	2	100
Dahinten et al., 2016 [15]	2	1	2	2	0	70
Manojlovich et al., 2002 [16]	2	2	2	2	2	100
Kretzschmer et al., 2017 [17]	2	1	2	2	0	70
Kostiwa & Meeks, 2009 [18]	2	2	2	2	2	100
Chang et al., 2010 [19]	2	1	2	2	0	70
Ding & Wu, 2023 [20]	2	2	2	2	0	80
Çankaya & Eriş, 2022 [21]	2	2	0	2	2	80
Ertem et al., 2012 [22]	2	1	2	2	0	70
Choi & Kim, 2019 [23]	2	1	0	2	2	70
Ahmad & Oranye, 2010 [24]	2	2	2	2	0	80
Engström et al., 2010 [25]	2	2	0	2	2	80
Rafiq et al., 2020 [26]	2	1	0	2	0	50
Orlowska & Laguna, 2023 [27]	2	1	1	2	2	80
Al-Hussein, 2020 [28]	2	2	2	2	0	80
Orgambídez & Almeida, 2020 [29]	2	1	2	2	0	70

Data Synthesis

I^2^ was utilized to assess the heterogeneity of studies relying on the random-effect model. I^2^ of 25% indicates low heterogeneity, 50% moderate, and 75% high heterogeneity. The % represents the proportion of variance in the total variance of the study population. Funnel plots with Begg’s rank and Egger’s weighted regression test were employed to assess the publication bias. Sensitivity analysis was utilized to measure the robustness of study results using the one-leave-out approach. The correlation coefficients transformed to Fisher’s Z and standard error were used to capture pooled r effect size with 95% confidence intervals (CIs). Data analysis was performed using Compressive Meta-Analysis Software (CMA) V. 4.0 (Englewood, NJ, USA).

Study Selection

**Figure 1:**
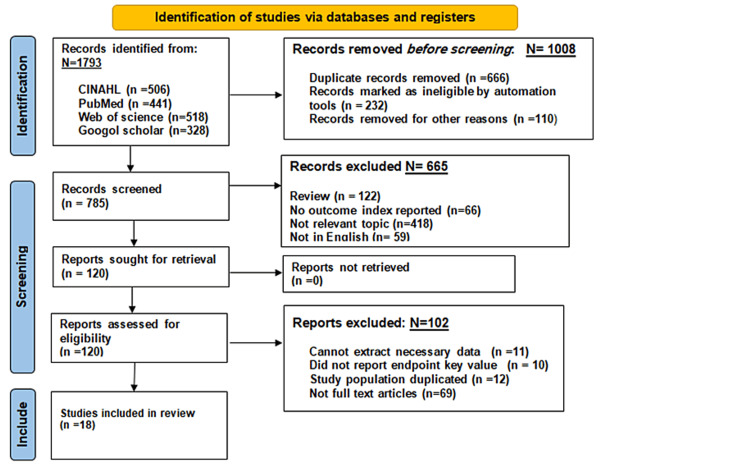
Preferred Reporting Items for Systematic Reviews and Meta-Analysis 2020 flow diagram.

Through database search, a total of 1,793 articles were retrieved. Before the screening stage, 1,008 articles were removed. The remaining 785 studies were assessed by titles and abstracts, resulting in the exclusion of 665 records. The remaining 120 studies were assessed for inclusion by reading the full text, resulting in the exclusion of 102 studies. Consequently, 18 articles that met the eligibility criteria were included in our review and analysis. The PRISMA flowchart presented in Figure 1 outlines the 18 studies that fulfilled the criteria.

Results

Study Characteristics

Eighteen eligible studies were included in the final analysis encompassing 6,353 registered nurses with ages ranging from 25 to 42 years. The included studies were conducted from 2001 to 2023. Four studies were from Canada [7,14-16], three from the United States [6,17,18], two studies from China [19,20], two from Turkey [21,22], and one each from South Korea [23], Malaysia and England [24], Sweden [25], Pakistan [26], Poland [27], Iraq [28], and Portugal [29]. The vast majority of studies used reliable and valid study tools, namely, the Psychological Empowerment Scale (PES) and the Job Satisfaction Scale (JSS) which showed a high Cronbach’s α coefficients. The correlation coefficients varied widely (*r* = -0.08 to 0.81). The highest correlation coefficient (*r* = 0.81) was found in the study by Kretzschmer et al. [17], while the least was found in the study by Laschinger et al. [7] (*r* = -0.08). Two studies showed an inverse weak relationship [6] with one non-significant [7], while three studies reported weak significant positive correlation [14,21,24]. Five studies reported moderate significant positive correlation [20,24,26-28]. Finally, nine studies reported a strong significant positive correlation [15-19,22,23,25,29] (Table 2).

**Table 2:** Characteristics of the included studies. WQI = Work Quality Index for job satisfaction; PES = Psychological Empowerment Scale; CWEQ = Conditions of Work Effectiveness for Empowerment; MMSS = Mueller/McCloskey Nurse Job Satisfaction Scale; JSS = Job Satisfaction Scale

First author and publication year	Country	Sample size of RN	Measures	Reliability Cronbach’s α	Statistical analysis	r coefficients	Strength and direction of correlation
Larrabee et al., 2003 [6]	United States	90	WQI, PES	0.95, 0.91	Correlation and multiple regression	-0.25	Weak and negative
Laschinger et al., 2004 [7]	Canada	286	JSS, PES	0.78–0.84, 0.87–0.89	Structural equation modeling	-0.08	Very weak and negative
Boamah et al., 2017 [14]	Canada	400	JSS, CWEQ-11	0.82, 0.85	Correlation and multiple regression	0.24	Weak and positive
Dahinten et al., 2016 [15]	Canada	1007	PES, Revised MMSS-25	0.78, 0.87	Pearson correlation and hierarchical linear regression	0.63	Strong and positive
Manojlovich et al., 2002 [16]	Canada	347	JSS, PES, CWEQ	0.81, 0.88, 0.95	Pearson correlation and hierarchical linear regression	0.62	Strong and positive
Kretzschmer et al., 2017 [17]	United States	484	JSS, CWEQ-11	0.95, 0.82	Correlation and multiple linear regression	0.81	Strong and positive
Kostiwa & Meeks, 2009 [18]	United States	56	PES, JSS	0.83–0.87, 0.93–0.94	Correlation and multiple regression	0.64	Strong and positive
Chang et al., 2010 [19]	China	330	PES, JSS, CWEQ-11	NA, 0.77, 0.89	Linear regression, path analysis, and structural equation modeling	0.66	Strong and positive
Ding & Wu, 2023 [20]	China	507	PES, JSS	0.73–0.76, 0.78	Correlation and structural equation modeling	0.54	Moderate and positive
Cankaya & Eris, 2022 [21]	Turkey	684	Career satisfaction, PES	0.90, 0.79	t-test, ANOVA, and correlation	0.32	Weak and positive
Ertem et al., 2012 [22]	Turkey	174	PES, JSS	0.72, NA	t-test, ANOVA, and correlation	0.69	Strong and positive
Choi & Kim, 2019 [23]	South Korea	208	CWEQ-11	0.86	Correlation and multiple regression	0.61	Strong and positive
Ahmad & Oranye, 2010 [24]	Malaysia	388	JSS	0.78–0.90	Spearman rho and multiple regression	0.33	Weak and positive
	England	168	JSS	0.90		0.57	Moderate and positive
Engstrom et al., 2010 [25]	Sweden	46	JSS, PES	NA, NA	Spearman rho correlation	0.60	Strong and positive
Rafiq et al., 2020 [26]	Pakistan	398	PES, JSS	0.79, 0.81	Structural equation modeling	0.51	Moderate and positive
Orlowska & Laguna, 2023 [27]	Poland	309	PES, JSS	0.89, 0.86	Multilevel modeling HLM	0.49	Moderate and positive
Al-Hussein, 2020 [28]	Iraq	317	PES, JSS	0.79, 0.83	Correlation and stepwise regression analysis	0.58	Moderate and positive
Orgambídez & Almeida, 2020 [29]	Portugal	124 RN + 30 certified nursing assistants	JSS, CWEQ-II	NA, NA	Hierarchical multiple linear regressions	0.63	Strong and positive

**Figure 2:**
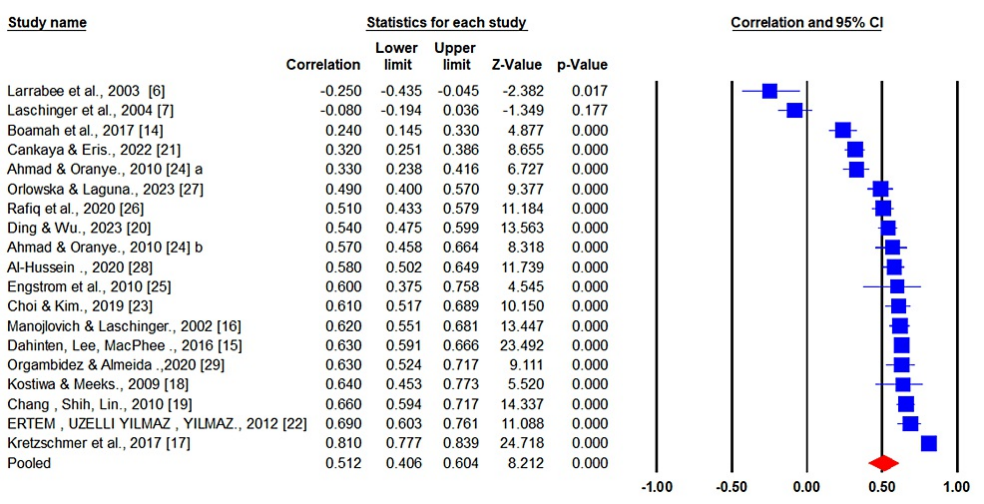
Forest plot assessing effect size.

In the same context, the forest plot showed that the pooled correlation between psychological empowerment and job satisfaction was0.512 (95% CI = 0.406-0.604), indicating that the association is moderate and positive based on the included studies in the meta-analysis. Furthermore, the heterogeneity among studies was mild to moderate, as suggested by I^2^ of 35% (Figure 2).

Publication Bias

**Figure 3:**
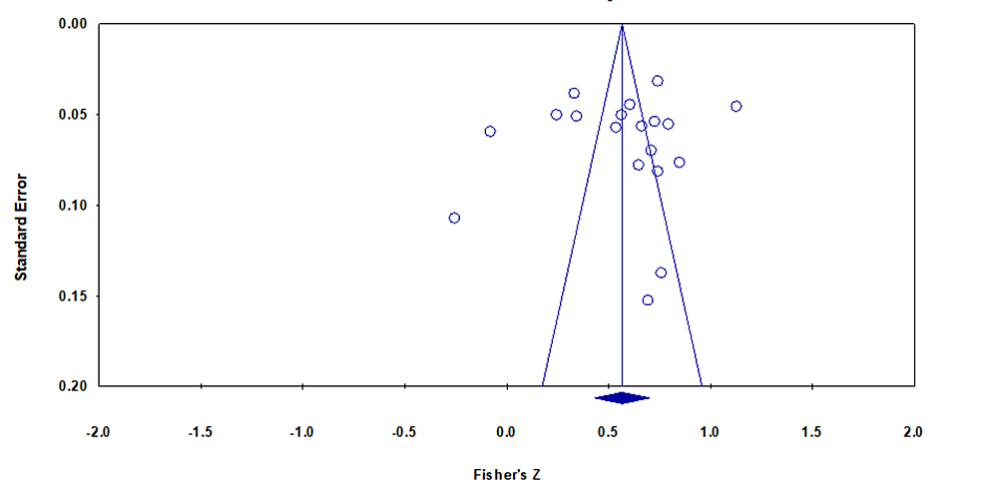
Funnel plot assessing publication bias.

According to the funnel plot shown in Figure 3, publication bias was detected, with most studies clustered into one part and unevenly distributed. Furthermore, Egger’s regression and Begg’s rank test showed a p-value <0.05.

Sensitivity Analysis

**Figure 4:**
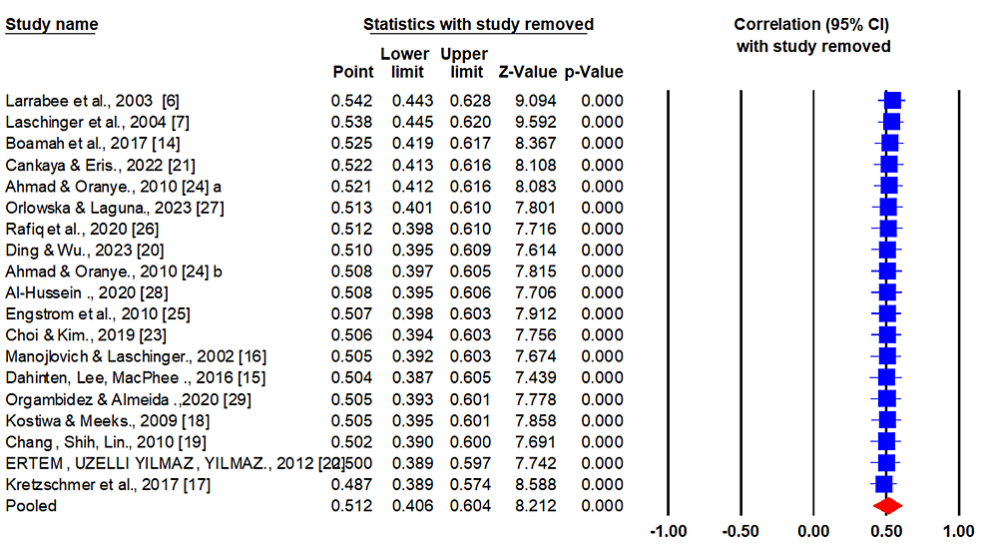
Sensitivity analysis using the one-leave-out method.

Sensitivity analysis allows the researcher to examine the extent to which the results of the meta-analysis might change when certain assumptions or decisions are altered. Sensitivity analysis revealed that the findings of the meta-analysis were robust even after removing one study (Figure 4).

Discussion

Psychological empowerment is a process involving the interplay between individuals’ internal personal traits and the workplace environment. Specifically, it includes four cognitive domains, namely, (1) matching work requirements and individuals’ beliefs, (2) self-efficacy to achieve tasks skillfully, (3) the feeling of having choice over one’s work autonomy and continuity of work activities in the workplace environment, and (4) perception of capacity to influence work outcome [30].

Previous research suggested that psychological empowerment can foster the interaction between the external environment and an individual’s belief, as psychological empowerment is considered an internal motivator [31]. This was confirmed by Conger & Kanungo (1988) who suggested that the sense of autonomy among employees can be enhanced by eliminating disempowering structures from the workplace environment which fosters loyalty to their work [32]. This perception of empowerment and work tendencies supports the theoretical proposition that feeling empowered is a mediator between workplace context and individual behavior [33-35].

Recently, two meta-analyses have assessed the relationship between psychological empowerment and job satisfaction among nurses. In the first study, 11 eligible articles were included in the final analysis. The pooled correlation coefficient between the two measured constructs was 0.353 (95% CI = 0.208-0.484) [8]. However, the current meta-analysis included more studies (n = 18) and revealed a higher pooled correlation coefficient of 0.512 (95% CI = 0.406-0.604). Additionally, our findings closely align with the findings of the second meta-analysis conducted by Gu et al. (2022), which showed the relationship between psychological empowerment and job satisfaction was 0.55 (95% CI = 0.53-0.56) based on 28 included studies [36]. These congruencies may suggest that the current work is reliable with robust results.

In this study, 17 studies revealed that psychological empowerment has a relationship with job satisfaction, or psychological empowerment is a contributing variable for job satisfaction, whereas one study failed to find this link between the two constructs [7]. Moreover, the results of this study (n = 16) demonstrated a positive correlation between the two measured constructs. Additionally, two studies investigated the relationship between structural empowerment and job satisfaction, as mediated by the role of psychological empowerment. The first study [7] failed to find the mediation role of psychological empowerment on the relationship between structural empowerment and job satisfaction. However, Dahinten et al. [15] found that psychological empowerment has a direct effect on job satisfaction, but after accounting for the effect of structural and leader empowerment, psychological empowerment was no longer effective. This finding contradicts the outcomes of the cognitive empowerment model which was developed in the West [33]. These contradictions can be attributed to the fact that nurses from different countries may interpret the concept of empowerment differently, as the concept originated in the West. For instance, in China, the concept of empowerment is understood as a dynamic complementary way to avoid conflicts with administrators [37], revealing that if managers fail to empower their employees, they will not have the psychological ability to do their jobs. Additionally, as these studies were performed in diverse settings, future approaches could examine the mediating role of psychological empowerment within consistent settings and contexts.

Finally, numerous studies have shown positive outcomes of empowerment. Conger & Kanungo (1988) suggested that empowerment can produce a positive effect if the individuals feel empowered, which, in turn, can also positively impact patients’ sense of empowerment [32]. Additionally, two studies pointed out that a high level of empowerment is linked with lower turnover and stress and increased workplace satisfaction and commitment, resulting in positive health outcomes [38,39]. Thus, triggering empowerment is crucial for nurses and patients.

Limitations

This work has included several limitations. First, all included articles were observational and cross-sectional constraining causality. Second, most studies were conducted in developed countries and a minority were conducted in developing countries. In addition, our findings may be affected by a population’s standard of living or the medical environment, further reducing the ability to generalize findings for all nurses worldwide. Third, the source of heterogeneity may be explained by the use of different study tools to measure psychological empowerment and job satisfaction. Fourth, the study instrument’s reliability was not reported in six studies, potentially impacting the accurate representation of the true relationship. Finally, the relationship between two measured constructs may affect other mediating or confounding variables. Therefore, investigating or controlling for these variables is highly recommended in future research in investigating the relationship between empowerment and job satisfaction.

Implication for clinical practice and future research

This work may assist hospital administrators in creating strategies to preserve an empowered workplace, which will, in turn, enhance nurses’ job satisfaction, reduce turnover, and improve patient safety. Moreover, this work may provide opportunities to conduct additional studies with different methodologies to iterate the nature of the relationship between psychological empowerment and job satisfaction.

## Conclusions

The meta-analysis provided important evidence that psychological empowerment has a reasonable association with nurse satisfaction. Hence, designed strategies should be adopted by administrators to enhance nurses’ psychological empowerment level to improve work satisfaction and reduce turnover. However, further longitudinal or experimental studies are required to capture the causal inferences.
